# Culturally Targeted Video Improves Psychosocial Outcomes in Latina Women at Risk of Hereditary Breast and Ovarian Cancer

**DOI:** 10.3390/ijerph16234793

**Published:** 2019-11-29

**Authors:** Alejandra Hurtado-de-Mendoza, Kristi D. Graves, Sara Gómez-Trillos, Pilar Carrera, Claudia Campos, Lyndsay Anderson, George Luta, Beth N. Peshkin, Marc D. Schwartz, Ana-Paula Cupertino, Nathaly Gonzalez, Vanessa B. Sheppard

**Affiliations:** 1 Department of Oncology, Georgetown University Medical Center, 3300 Whitehaven Street, Suite 4100, Washington, DC 20007, USA; Kristi.Graves@georgetown.edu (K.D.G.); sg1328@georgetown.edu (S.G.-T.); gl77@georgetown.edu (G.L.); peshkinb@georgetown.edu (B.N.P.); Schwartm@georgetown.edu (M.D.S.); 2 Jess and Mildred Fisher Center for Hereditary Cancer and Clinical Genomics Research, 3300 Whitehaven Street, Suite 4100, Washington, DC 20007, USA; 3 Department of Social Psychology and Methodology, Universidad Autónoma de Madrid, 28049 Madrid, Spain; pilar.carrera@uam.es; 4 Nueva Vida, DC Office—801 N Pitt St., Suite 113, Alexandria, VA 22314, USA; ccampos@nueva-vida.org; 5 College of Health and Human Services, School of Nursing, California State University, Sacramento, CA 95819, USA; ltw7@georgetown.edu; 6 Department of Biostatistics, Bioinformatics and Biomathematics, 4000 Reservoir Rd., NW, Washington, DC 20057, USA; 7 Cancer Prevention and Control Program, John Theurer Cancer Center, Hackensack University Medical Center, 40 Prospect Avenue, Office number 316, Hackensack, NJ 07601, USA; Paula.Cupertino@hackensackmeridian.org; 8 Capital Breast Care Center, 1000 New Jersey Ave, SE, Washington, DC 20003, USA; ng472@georgetown.edu; 9 Department of Health Behavior Policy, Virginia Commonwealth University, Richmond, VA 23298, USA; Vanessa.Sheppard@vcuhealth.org; 10 Massey Cancer Center, Office of Health Equity and Disparities Research, Richmond, VA 23298, USA

**Keywords:** hereditary breast and ovarian cancer, Latinos, disparities, intervention

## Abstract

Latina women at risk of hereditary breast and ovarian cancer (HBOC) have lower awareness, knowledge, and use of genetic counseling and testing services (GCT) than non-Latina Whites. Few interventions have been developed to reduce these disparities among at-risk Latinas. This pilot study assessed the impact of a culturally targeted narrative video developed by our team. The study included 40 Latina immigrants living in the United States who were at risk of HBOC, including affected and unaffected women. We assessed pre-post differences in psychosocial outcomes. Participants were 47.35 years old on average (SD = 9.48). Most (70%) were unaffected with cancer, had an annual income of $40,000 or less (65%), an education of High School or less (62.5%), and were uninsured (77.5%). The video significantly enhanced knowledge (*p* < 0.001), positive attitudes (*p* < 0.05), anticipatory positive emotions (*p* < 0.05), and intentions to participate in counseling (*p* < 0.001). Importantly, the video also significantly reduced negative attitudes (*p* < 0.05), and attitudinal ambivalence (*p* < 0.001). The culturally targeted video shows preliminary evidence in improving psychosocial outcomes related to GCT uptake in Latinas at risk for HBOC. This intervention is a promising easily-disseminable strategy to address disparities in GCT utilization.

## 1. Introduction

*BRCA1/2* gene mutations are the most commonly identified mutations that increase the risk of hereditary breast and ovarian cancer (HBOC) [1]. Women with the mutation (carriers) have up to 70% and up to 40% lifetime risk of developing breast and ovarian cancer, respectively [2,3,4]. Breast cancer survivors with *BRCA1/2* mutations are three times more likely to develop contralateral breast cancer than non-carriers [5]. National guidelines recommend referral to genetic cancer risk assessment for all women at high risk of HBOC based on personal and family history [6,7]. Genetic cancer risk assessment typically includes meeting with a genetic counselor and considering the option of genetic testing if recommended. A positive *BRCA1/2* gene test result impacts treatment choices in women diagnosed with cancer and risk management in women with and without a cancer diagnosis [8], including an uptake of risk-reducing surgeries [9]. Risk-reducing surgeries can reduce breast cancer risk by over 90%, ovarian cancer risk by 85–90% [10,11], and increase life expectancy among mutation carriers [12]. 

Breast cancer is the most commonly diagnosed cancer and the leading cause of cancer death in Latinas [13]. Among Latinas in the U.S., the population prevalence of *BRCA1/2* mutations is at least double that of the general population. [14] Despite their high mutation prevalence, Latinas are significantly less likely to participate in genetic counseling and testing (GCT) compared to NHW [15,16,17,18]. Underuse of GCT limits Latinas’ access to information that can inform their risk management and treatment choices, thereby improving their health outcomes [15]. 

Disparities in GCT awareness are pervasive [19]. In a population registry from the National Health Interview Survey, the percentage of Latinos (21%) who reported awareness of cancer genetic testing in the year 2000 was lower than Whites (50%). This gap remained stable over the 2000–2010 decade [20]. Studies have also found low GCT awareness among Latinos at risk of HBOC [21,22,23]. Awareness is even lower among Latinos with low education, low English proficiency, low acculturation, and those who are foreign born [24,25]. Additionally, Latinos have low to moderate knowledge about HBOC and GCT [19] and lower GCT knowledge compared to NHW [26,27]. Thus, developing interventions to increase awareness about the availability of GCT services and knowledge about HBOC risk in Latinos is a key step to reducing persistent gaps in awareness and GCT utilization.

Ensuring that complex genetic information is understandable and culturally appropriate is critical, especially for the most vulnerable subgroup of Latina immigrants with low education, low health literacy, and limited English proficiency. Substantial evidence documents at-risk Latinas’ preference for interventions and education materials in Spanish that include plain language, visual aids, and a narrative format [27,28,29]. Evidence suggests that narratives can be an effective strategy for cancer-related communication, especially for populations with low education and health literacy [30,31,32]. Studies conducted with Latinos suggest that narrative interventions are more effective than non-narrative interventions in enhancing outcomes related to cervical cancer screening [33], breast cancer screening [32], and colorectal cancer screening [34]. For instance, a culturally narrative film produced greater increases in cervical cancer knowledge and higher uptake of cervical screening compared to a non-narrative film. Importantly, the culturally targeted narrative film eradicated baseline disparities in Pap smear uptake between Mexican Americans and non-Hispanic Whites [33]. 

We developed a culturally targeted Spanish-language narrative video for at-risk Latinas. The development process is reported elsewhere [35]. Briefly, our multidisciplinary team collaborated with filmmakers from an actor training studio program to develop the script and video. The 18 min video illustrates the GCT process with the story of Rosa, a Latina breast cancer survivor who learns about her risk for HBOC, overcomes barriers to attend genetic counseling, and attends a genetic counseling appointment. The script was informed by extensive formative research, evidence-based risk communication strategies, and health behavior models [35]. For instance, the video sought to elicit emotions, to convey information to address knowledge gaps (e.g., age at breast cancer diagnosis as a risk factor), and to clarify misconceptions identified in our formative research (e.g., the misconception that Pap smear is a screening test for ovarian cancer) [35,36]. The video also targeted psychosocial outcomes from the Theory of Planned Behavior (TPB) [37], including attitudes, subjective norms, perceived control, and behavioral intentions.

In addition to TPB constructs, we targeted other constructs that have emerged from prior literature. Prior research suggests that once informed, Latinos tend to hold positive attitudes towards GCT [28,29,38]. However, studies also show that Latinos have negative attitudes related to concerns about cost, cancer stigma, discrimination, and anticipation of negative emotions [39]. This suggests that Latinos may feel ambivalent towards GCT (holding both positive and negative attitudes) [40], which reduces the association between the TPB constructs of attitudes, intentions, and behaviors [41,42]. Although not always included in the TPB, anticipatory emotions (emotions felt right now when thinking about a future behavior) [43,44] are powerful determinants of health behaviors, and they have proven to enhance the predictive capacity of health behavior models [43,45,46]. Emotional concerns about GCT include fear and worry about obtaining a positive test result, guilt about passing a mutation to children [21,22,36,39,47], and may also include shame toward obtaining a positive result [39]. 

This paper presents psychosocial outcomes from a pre-post pilot study of the culturally targeted video described above. Guided by the key psychosocial variables within the expanded TPB, we assessed change in knowledge as a primary outcome and change in attitudes, subjective norms, perceived control, behavioral intentions, anticipatory emotions, and ambivalence as secondary outcomes. We hypothesized that, after viewing the video, women would exhibit increased knowledge, subjective norms, perceived control, and behavioral intentions toward GCT and more positive attitudes compared to their reports immediately before watching the video. We also hypothesized that watching the video would enhance favorable anticipatory emotions and reduce ambivalence towards participating in GCT. The goals of this paper are to (1) report immediate pre-post differences in knowledge and other psychosocial outcomes and (2) explore whether education and health literacy were related to changes in knowledge.

## 2. Materials and Methods

### 2.1. Procedures

Our primary recruitment method was through partnerships with two community-based organizations (CBOs) that provide patient navigation services to Latinas in the mid-Atlantic area of the U.S. Additionally, we conducted community outreach via flyers and radio, and contacted women from a research registry who had agreed to be contacted for future studies [48]. In the CBOs, trained patient navigators conducted an initial assessment to identify potentially eligible participants and provided a brief explanation about the study. Women who were interested provided permission for the patient navigator to share their contact information with bilingual/bicultural Research Assistants (RAs) from the study team. RAs conducted a second screening to determine eligibility, provided more details about the study, and scheduled an in-person meeting either at the CBOs or at the university. Participants: Women were eligible if they self-identified as Latina/Hispanic, spoke Spanish fluently, were 18 years old or older, met 2018 National Comprehensive Cancer Network (NCCN) guidelines [49] for HBOC genetic counseling referral, and had never received genetic counseling nor testing. Both affected and unaffected women were eligible.

#### Video Intervention and Assessment 

At the in-person meeting, the RAs obtained participants’ verbal consent and administered verbally a pre-video baseline survey that lasted between 20–30 min. All assessments were completed individually, in a private room, and in Spanish. After completing the pre-video survey, participants watched the 18 min video. Immediately after the video, the RAs administered verbally a post-intervention survey that lasted between 20–30 min. Participants received a $30 gift card. Interested participants were referred to free or low cost GCT through patient navigators at our partner CBOs. The Georgetown University Institutional Review Board approved all the study procedures. 

### 2.2. Measures

#### 2.2.1. Sociodemographic and Clinical Factors

We assessed sociodemographic factors in the pre-video survey, including age, race, country of birth, years of education, marital status, income, and insurance status. Participants answered Yes/No to “In the past year, have you needed to visit a doctor, but could not go because of cost?”, and also answered “How difficult is it for you/your family to meet monthly bill payments?” with response options ranging from 1—not at all difficult to 5—extremely difficult [50]. We assessed health literacy with one item on a 5-point Likert scale [51] that asked participants to report self-confidence in filling out medical forms by themselves. Responses ranged from 1—not at all confident to 5–extremely confident. Self-rated Health: We assessed self-rated health with a widely used item from CDC’S Behavioral Risk Factor Surveillance System (BRFSS) questionnaire in which participants were asked to rate their general health. Response options included excellent, very good, good, fair, and poor [52]. Clinical factors included breast or ovarian cancer diagnosis (Yes/No). Women affected with cancer answered follow-up questions about their age and stage at diagnosis.

#### 2.2.2. GCT Awareness and Referrals

We used a 4-item scale to assess GCT awareness [29]. Participants rated on a 4-point Likert-type response scale how much they had heard or read about genetic counseling for hereditary diseases, for cancer in general, for breast cancer, and for ovarian cancer (1—almost nothing; 4—very much). Higher scores represent higher genetic counseling awareness. Participants also responded whether they had ever been referred by a doctor to genetic counseling (Yes/No) and to genetic testing (Yes/No).

#### 2.2.3. Psychosocial Outcomes 

We assessed psychosocial outcomes immediately before and after the video, including knowledge, behavioral intentions, attitudes, perceived control, subjective norms, anticipatory emotions, and attitudinal ambivalence.

##### Primary Outcome

Knowledge: We measured three distinct types of knowledge. We assessed breast cancer genetics knowledge with Erblich and colleagues’ (2005) [53] validated 13-item scale about breast cancer genetics. We assessed knowledge of genetic counseling with Kasting and colleagues’ 9-item scale [54]. We developed a 20-item scale that tapped into the main informational messages about HBOC delivered in the video (e.g., HBOC risk factors, purpose of GCT) (α = 0.49 pre; α = 0.61 post). Response options for all three scales included True/False/I don’t know. We added the number of correct responses to create a score for each of the scales. Higher scores mean higher knowledge. 

##### Secondary Outcomes

Behavioral Intentions: We used two items validated with this population to assess participants’ intentions/likelihood of having genetic counseling and testing, respectively, in the next three months with response options ranging from 1—not at all likely to 5—extremely likely [29]. 

Based on recommended guidelines [55] we developed a scale to capture other TPB constructs, attitudinal ambivalence, and anticipatory emotions with items on a 7-point Likert-type response (from 1—strongly disagree to 7—strongly agree) that referred to the specific behavior of participating in genetic counseling in the next three months.

Attitudes: Positive attitudes were assessed with two items (i.e., Participating in genetic counseling in the next three months would be good/useful; α = 0.77 pre and post) on a 7-point Likert-type response (from 1—strongly disagree to 7—strongly agree). Higher scores indicated higher favorable attitudes. Negative attitudes were measured with two items (i.e., Participating in genetic counseling in the next three months would be bad/not useful”; α = 0.84 pre and α = 0.63 post) on a 7-point Likert-type response (from 1—strongly disagree to 7—strongly agree). Higher scores indicated higher negative attitudes.

Perceived control: Perceived control was measured with two items on a 7-point Likert-type response (from 1—strongly disagree to 7—strongly agree) (i.e., I feel confident about participating in GCT; participating in GCT depends on me). Higher scores indicated higher perceived control. Since the alpha was low, we analyzed each item separately (α = 0.75 pre and α = 0.30 post).

Subjective Norms: Subjective norms were measured with two items on a 7-point Likert-type response (from 1—strongly disagree to 7—strongly agree). Injunctive norms evaluated to what extent loved ones approve that participants receive genetic counseling in the next three months (“*The people I care about would approve of me going to GCT*”) while descriptive norms measured participants’ beliefs about genetic counseling uptake of the majority of people like them *(*“*Most people like me (at-risk of HBOC) receive GCT*”). Higher scores indicated higher injunctive and descriptive norm, respectively.

Attitudinal Ambivalence: We measured attitudinal ambivalence with two items on a 7-point Likert-type response (from 1—strongly disagree to 7—strongly agree). One item captured the cognitive aspect of attitudes (i.e., I have doubts), and the other item measured the affective aspects of attitudes (i.e., I have bittersweet feelings). Higher scores indicated higher cognitive and affective ambivalence, respectively.

Anticipatory Emotions: Participants rated to what extent they felt six different positive emotions ‘right now’ (e.g., calm) and six negative emotions (e.g., fear) when thinking about participating in genetic counseling in the next three months on a 7-point Likert-type response (from 1—strongly disagree to 7—strongly agree). Following prior studies [43,56,57], we calculated for each participant the mean of the positive anticipatory emotions (α = 0.88 and 0.83 in pre and post video, respectively) and the mean of the six negative anticipatory emotions (α = 0.82 and 0.73 in pre and post video, respectively). 

### 2.3. Statistical Analysis

We used descriptive statistics to summarize the sociodemographic data, and clinical data, genetic counseling awareness, and genetic counseling/testing referrals. We conducted paired t-tests to evaluate changes (from before the video to after the video) in the psychosocial factors. To examine changes in the proportion of correct responses on the items from the main information knowledge scale, we conducted a binomial Mc Nemar test. To assess whether changes in the three knowledge scales varied by education and health literacy, we stratified the sample into participants with low vs. high education (High School or below (n = 25) vs. some college and more (n = 15) and low (n = 16) vs. high (n = 24) health literacy (participants reporting feeling not comfortable/kind of comfortable vs. pretty/really/extremely comfortable filling medical forms by themselves). Then conducted paired t-tests to evaluate changes in knowledge separately for each stratum. The significance threshold was set at *p* < 0.050. All statistical analyses were performed using SPSS 25 [58]. 

## 3. Results

Participants had an average age of 47.35 (SD = 9.48), and most were born in El Salvador (52.5%), followed by Peru (15%). Most were married (60%), had an education of High School or lower (62.5%), annual household income of $40,000 or lower (65%), and were uninsured (77.5%). Additionally, 40% reported feeling not comfortable/kind of comfortable completing medical forms by themselves. Most were unaffected with cancer (70%). Among the 12 women affected with cancer, 10 were diagnosed with breast cancer and two with ovarian cancer. Awareness was very low as 83.9%, and 83.3% of participants had heard almost nothing or relatively little about genetic counseling for hereditary breast cancer and for hereditary ovarian cancer risk, respectively. No participants had been referred to genetic counseling, and only 10% had been referred to genetic testing but had never received the genetic testing (see Table 1).

### 3.1. Pre-Post Video Differences

#### 3.1.1. Main Outcome 

Knowledge levels were low to moderate before the video. The estimated mean differences between pre and post are denoted by “*D*.” After the video, knowledge significantly increased in the three distinct knowledge measures including breast cancer genetics knowledge (*D* = 2.05, t (37) = 6.81, *p* < 0.001), genetic counseling knowledge (*D* = 0.89, t (38) = 3.78, *p* = 0.001), and the main information knowledge scale (*D* = 4, t (34) = 9.26, *p* < 0.001) (see Table 2). The video changed key misconceptions and knowledge gaps identified in our research. For instance, before watching the video, the proportion of participants who held the misconception that Pap smear was a screening test for ovarian cancer (80% incorrect responses) significantly reduced (15% incorrect responses) after watching the video (*p* < 0.001). Also, the proportion of the sample (50% correct responses) who correctly identified breast cancer diagnosis at 50 or younger as a risk factor of HBOC increased significantly (95% correct responses) after the video (*p* < 0.001). Additionally, the three knowledge measures significantly increased in women with low and high education and in women with low and high health literacy (see Table 3).

#### 3.1.2. Secondary Outcomes

TPB Outcomes: Participants had high ratings for most TPB variables (i.e., positive attitudes, behavioral intentions, injunctive norms, and perceived control) before watching the video, potentially creating a ceiling effect for some of the variables. *Attitudes*: lthough participants held initial positive attitudes, watching the video significantly increased favorable attitude towards receiving genetic counseling (*D* = 0.23, t (39) = 2.12, *p* = 0.04) and significantly reduced negative attitudes (*D* = −0.43, t (36) = −2.33, *p* = 0.02). *Behavioral intentions:* The video significantly enhanced intentions to participate in counseling (*D* = 0.51, t (38) = 3.62, *p* = 0.001) but there were no statistically significant differences in intentions to participate in genetic testing (*D* = 0.03, t (38) = 0.21, *p* = 0.84). 

Subjective norms: The video increased the injunctive norm approaching significance (*D* = 0.16, t (37) = 1.97, *p* = 0.057). Descriptive norm presented a similar significance tendency (*D* = 0.59, t (36) = 1.71, *p* = 0.09). *Perceived control:* Participants did not significantly change their confidence to be able to go to a genetic counseling session after watching the video (*D* = 0.15, t (39) = 0.68, *p* = 0.50). A non-significant result was also obtained when we evaluated how much going to a genetic counseling session in the next three months depended on them (*D* = 0.12, t (39) = 1.00, *p* = 0.32) (see Table 2). 

Ambivalence: Before the video participants had moderate ratings of ambivalence, including having doubts (cognitive ambivalence) and mixed feelings (affective ambivalence) towards participating in counseling. The video significantly reduced both the cognitive (*D* = −1.10, t (38) = −2.62, *p* = 0.012) and the affective ambivalence (*D* = −0.85, t (38) = −2.02, *p* = 0.050) (see Table 2).

Anticipatory Emotions: Despite having high initial ratings of positive anticipatory emotions, watching the video significantly improved the positive anticipatory emotions (*D* = 0.22, t (39) = 2.10, *p* = 0.04) while the negative emotions remained low and similar (*D* = −0.30, t (38) = −1.54, *p* = 0.13) (see Table 2). The positive anticipatory emotions that showed statistically significant differences were relief (*D* = 0.35, t (39) = 2.16, *p* = 0.037) and pride (*D = 0.37*, t (39) = 2.56, *p* = 0.014). Hopefulness approached significance (*D = 0.27*, t (39) = 1.98, *p* = 0.054).

## 4. Discussion

Our culturally targeted narrative video constitutes one of the few psychoeducational interventions about HBOC targeted to Spanish-preferring, at-risk Latina women [29,59,60]. The video showed promising findings in improving knowledge and other important psychosocial outcomes including attitudes, anticipatory positive emotions, ambivalence, and intentions to participate in genetic counseling. 

At-risk Latina women are significantly less likely to use GCT services compared to NHWs [16,17,18]. Increasing awareness about GCT and HBOC risk is a logical first step in closing this gap. Similar to prior studies [21,22,23,29] and despite being an at-risk population, our sample had extremely low awareness and low to moderate levels of knowledge at baseline. Watching our culturally-targeted video had a large and robust effect on knowledge gains reflected by three distinct measures. This is important, given the documented associations between knowledge and GCT uptake [61,62,63]. Importantly, participants with low education and low health literacy also experienced significant increases in knowledge. This finding supports prior studies, which suggest that the use of narratives is a particularly effective way of conveying health information for populations with low health literacy [33,51,64]. Our culturally targeted video has the potential to raise awareness and knowledge of a complex health topic in a large population of Latinos that are especially vulnerable. 

In addition to knowledge gains, our video enhanced other psychosocial outcomes that have been associated with GCT uptake [19]. Although most participants were unaware of genetic counseling at baseline, similar to prior studies [19,21], most participants reported favorable attitudes when they received a brief explanation. Despite a noticeable ceiling effect on positive attitudes, positive anticipatory emotions, and intentions to participate, the significant increase in these constructs suggests that the change may be potentially due to an increase in tangible knowledge about GCT and HBOC risk, rather than potentially due to social desirability bias. 

Study participants had moderate levels of ambivalence at baseline (i.e., I have doubts; I feel bittersweet about participating in GCT), but watching the video significantly reduced ambivalence towards GCT participation. It is important to highlight that the relationship between attitudes-intentions-behavior is weaker for people who score high on ambivalence [41,42]. The positive effects that watching the video had on reducing cognitive ambivalence are indicative that the increase in knowledge diminished the doubts about GCT. In the affective pole, the decreased of mixed feelings could be explained by the changes in anticipatory emotions after the narrative; the video generated an increase of positive emotions and did not vary the negative effects; this differential influence could have reduced the affective ambivalence. 

The emotional reactions after the video are important, considering that the affective components are especially relevant when studying health-risk behaviors [65]. Future-oriented emotions constitute important proximal antecedents of behaviors associated with health [66,67]. Our positive results on emotional reactions constitute a hopeful way to promote GCT. Perceived threat is associated with defense mechanisms such as avoidance, denial, or reactance that reduce the efficacy of the message [68]. The video did not produce intense unpleasant feelings or extreme negative attitudes, a result that facilitates to attend the recommendations included in the message. The decrease of the level of ambivalence (cognitive and affective), in addition to the increase in knowledge, favorable attitudes, intentions, and anticipatory emotions, suggest that the video can help participants develop stronger and more favorable reactions towards GCT. 

Results from this study also highlight the potential of the video to target historically identified barriers that Latinas in the U.S face for reception of GCT, including language barriers and suboptimal referrals [16,21,47,69]. Few education materials are available in Spanish, and very few genetic counselors speak Spanish in the US [70,71]. Spanish-speaking Latinas diagnosed with breast cancer are almost five times more likely to report unmet needs for genetic testing discussion with their providers compared to their non-Latina counterparts [72]. In our sample of at-risk Latina women, only 10% were referred to genetic testing, and none had been referred to genetic counseling. Our culturally targeted video in Spanish can reach the U.S Latino Spanish-preferring population that has the largest information needs and may be able to prompt conversations with providers. Additionally, our video can be easily disseminated through the Internet, and it can be viewed across various platforms, including computers and mobile devices, thus multiplying the potential reach to underserved Latinos. 

### Strengths and Limitations

This culturally targeted narrative video constitutes one of the few psychoeducational interventions about HBOC targeted to at-risk Latina women. Our video contributes to and expands prior interventions for at-risk Latinas such as flip charts, or presentations that had only been piloted in small focus groups [13,16]. We piloted the video in an underrepresented sample of 40 Latina immigrants and assessed a novel and comprehensive list of psychosocial outcomes, including constructs such as ambivalence and anticipatory emotions.

Despite the strengths, the study also has some limitations. The small sample size precluded us from conducting multivariate analysis and may have impacted the power to detect significant differences. Future studies with larger samples are warranted. The completion of the pre and post surveys at the same study visit could have resulted in a response burden. However, the use of a less cognitive burdensome mode of administration (in-person, interviewer administered, in participants’ native language) [73] and the fact that all participants finished the pre and post surveys and no concerns about survey length were raised minimizes the potential for participant overburden. The psychosocial outcomes were only measured before and immediately after the video. Thus, future studies should include a longer follow-up to assess if changes in psychosocial outcomes are maintained. Six of the 40 participants were related (one pair of sisters and two mother–daughter pairs). Although related, participants completed the study at the same time, individually in different rooms, and the pairs of relatives had no contact with each other during the study procedures. However, it may still be possible for the changes in the outcomes of the related participants to be correlated. Due to the exploratory nature of this pilot study, we have not performed an adjustment for multiple testing. The study was conducted with a small convenience sample of Latina immigrants from Central and South America recruited from urban settings. Thus, findings may not generalize to other Latino populations from rural settings, from other regions (e.g., Caribbean), or to second-generation Latinas. Although we found preliminary evidence of the video in improving psychosocial outcomes, the study did not include a comparison arm. Future studies should evaluate the efficacy of the video in a larger randomized controlled trial. Future studies should also examine the best strategies to implement the video in clinical practice if proven to be efficacious as well as the best strategies for dissemination. 

## 5. Conclusions

Our culturally targeted educational video can help overcome several barriers that Latinos face to participation in GCT. Given the scarcity of materials in Spanish, low awareness of GCT, and limited knowledge, our easy-to-disseminate video represents a promising strategy to reduce disparities in GCT uptake.

## Figures and Tables

**Table 1:** Descriptive statistics (n = 40).

Sociodemographic and Clinical Factors	M(SD)
**Age**	47.35 (9.48)
	N (%)
**Race**	
White	7 (17.5)
More than one race	5 (12.5)
Other	22 (55.0)
Unknown	6 (1.0)
**Country of Origin**	
El Salvador	21 (52.5)
Peru	6 (15.0)
Guatemala	4 (10.0)
Mexico	4 (10.0)
Bolivia	3 (7.5)
Chile	1 (2.5)
Venezuela	1 (2.5)
**Marital Status**	
Married/Living as married	24 (60)
Divorced/Separated/Widowed/Never Married	16 (40)
**Education Level**	
High school or less	25 (62.5)
Some college and more	15 (37.5)
**Annual Salary**	
$40,000 or less	26 (65)
More than $40,000	10 (25)
Preferred not to answer/Missing	4 (10)
**Difficulty Paying Monthly Dues**	
Not at all/a little difficult	29 (72.5)
Very/extremely difficult	11 (27.5)
**Health Insurance**	
Insured	9 (22.5)
Uninsured	31 (77.5)
**Financial Burden-Health Visits**	
Yes	23 (57.5)
No	17 (42.5)
**Self-Reported Health**	
Very Good	3 (7.5)
Good	10 (25.0)
Normal	19 (47.5)
Poor	7 (17.5)
Prefer not to answer	1 (2.5)
**Cancer Diagnosis**	
Affected (breast/ovarian)	12 (30)
Unaffected	28 (70)
**Health Literacy-completing medical forms**	
Low: Not /kind of comfortable	16 (40)
High: Pretty/really/extremely comfortable	24 (60)
**GC Awareness for hereditary diseases (n = 31)**	
Almost nothing/Relatively Little	25 (80.6)
A fair amount/a lot	6 (19.4)
**GC Awareness for Cancer (n = 31)**	
Almost nothing/Relatively Little	24 (77.4)
A fair amount/a lot	7 (22.6)
**GC Awareness for Breast Cancer (n = 31)**	
Almost nothing/Relatively Little	26 (83.9)
A fair amount/a lot	5 (16.1)
**GC Awareness for Ovarian Cancer (n = 30)**	
Almost nothing/Relatively Little	25 (83.3)
A fair amount/a lot	5 (16.7)
**Genetic Counseling Referral (n = 31)**	
Yes	0 (0)
No	31 (100)
**Genetic testing Referral (n = 30)**	
Yes	3 (10)
No	27 (90)

**Table 2:** Pre-post changes in psychosocial outcomes.

Outcomes	Pre M (SD)	Post M (SD)	*p* Values
**Knowledge**			
Knowledge-Main Information Messages 20 items (0–20)	11.00 (2.54)	15.00 (2.24)	*p* < 0.001
Genetic Counseling Knowledge 9 items (0–9)	4.00 (1.72)	4.90 (1.55)	*p* = 0.001
Breast Cancer Genetics Knowledge 13 items (0–13)	5.08 (1.81)	7.13 (1.93)	*p* < 0.001
**Intentions**			
Intentions-GC (1–5)	4.18 (1.17)	4.69 (0.61)	*p* = 0.001
Intentions-GT (1–5)	4.56 (0.82)	4.59 (0.75)	*p* = 0.84
**Attitudes**			
Positive Attitudes (1–7)	6.46 (0.76)	6.70 (0.74)	*p* = 0.04
Negative Attitudes (1–7)	1.88 (1.49)	1.44 (0.76)	*p* = 0.025
**Norms**			
Injunctive Norm (1–7)	6.66 (0.58)	6.82 (0.48)	*p* = 0.057
Descriptive Norm (1–7)	4.03 (2.33)	4.62 (2.46)	*p* = 0.09
**Perceived Control**			
Confident (1–7)	6.25 (1.21)	6.40 (1.21)	*p* = 0.50
Depend on me (1–7)	6.38 (1.00)	6.50 (1.04)	*p* = 0.32
**Anticipatory Emotions**			
Positive emotions (1–7)	6.22 (0.89)	6.44 (0.77)	*p* = 0.042
Negative emotions (1–7)	2.14 (1.31)	1.84 (0.98)	*p* = 0.13
**Ambivalence**			
Cognitive ambivalence (1–7)	3.46 (2.28)	2.36 (1.83)	*p* = 0.012
Affective ambivalence (1–7)	4.46 (2.26)	3.62 (2.32)	*p* = 0.050

**Table 3:** Pre-post knowledge changes stratified by education and health literacy.

Outcomes	Pre *M*(*SD*)	Post *M*(*SD*)	*p*-Value
**Education**			
Low education			
Knowledge-Main Information Messages 20 items (0–20)	10.96 (2.32)	14.35 (2.19)	*p* < 0.001
Genetic Counseling Knowledge 9 items (0–9)	3.72 (1.90)	4.52 (1.45)	*p* = 0.017
Breast Cancer Genetics Knowledge 13 items (0–13)	5.00 (1.59)	6.87 (1.70)	*p* < 0.001
**High education**			
Knowledge-Main Information Messages 20 items (0–20)	11.08 (3.03)	16.25 (1.82)	*p* < 0.001
Genetic Counseling Knowledge 9 items (0–9)	4.50 (1.22)	5.57 (1.55)	*p* = 0.013
Breast Cancer Genetics Knowledge 13 items (0–13)	5.21 (2.19)	7.57 (2.28)	*p* = 0.001
**Health Literacy**			
**Low health literacy**			
Knowledge-Main Information Messages 20 items (0–20)	10.92 (2.36)	14.38 (2.22)	*p* = 0.001
Genetic Counseling Knowledge 9 items (0–9)	3.25 (1.29)	4.25 (1.34)	*p* = 0.006
Breast Cancer Genetics Knowledge 13 items (0–13)	5.07 (1.58)	6.67 (1.40)	*p* < 0.001
**High health literacy**			
Knowledge-Main Information Messages 20 items (0–20)	11.04 (2.70)	15.36 (2.21)	*p* < 0.001
Genetic Counseling Knowledge 9 items (0–9)	4.52 (1.80)	5.35 (1.55)	*p* = 0.025
Breast Cancer Genetics Knowledge 13 items (0–13)	5.09 (1.97)	7.43 (2.19)	*p* < 0.001
